# The Impact of Colony Deployment Timing on *Tetragonula carbonaria* Crop Fidelity and Resource Use in Macadamia Orchards

**DOI:** 10.3390/plants14152313

**Published:** 2025-07-26

**Authors:** Claire E. Allison, James C. Makinson, Robert N. Spooner-Hart, James M. Cook

**Affiliations:** Hawkesbury Institute for the Environment, Western Sydney University, Locked Bag 1797, Penrith, NSW 2751, Australia; j.makinson@westernsydney.edu.au (J.C.M.); r.spooner-hart@westernsydney.edu.au (R.N.S.-H.); james.cook@westernsydney.edu.au (J.M.C.)

**Keywords:** stingless bees, bee pollination, colony introduction timing, colony crop fidelity, foraging behavior, pollen collection, pollen diversity

## Abstract

Crop fidelity is a desirable trait for managed pollinators and is influenced by factors like competing forage sources and colony knowledge of the surrounding environment. In European honey bees (*Apis mellifera* L.), colonies deployed when the crop is flowering display the highest fidelity. We tested for a similar outcome using a stingless bee species that is being increasingly used as a managed pollinator in Australian macadamia orchards. We observed *Tetragonula carbonaria* (Smith) colonies deployed in macadamia orchards at three time points: (1) before crop flowering (“permanent”), (2) early flowering (“early”), and (3) later in the flowering period (“later”). We captured returning pollen foragers weekly and estimated crop fidelity from the proportion of macadamia pollen they collected, using light microscopy. Pollen foraging activity was also assessed via weekly hive entrance filming. The early and later introduced colonies initially exhibited high fidelity, collecting more macadamia pollen than the permanent colonies. In most cases, the permanent colonies were already collecting diverse pollen species from the local environment and took longer to shift over to macadamia. Pollen diversity increased over time in all colonies, which was associated with an increase in the proportion of pollen foragers. Our results indicate that stingless bees can initially prioritize a mass-flowering crop, even when flowering levels are low, but that they subsequently reduce fidelity over time. Our findings will inform pollinator management strategies to help growers maximize returns from pollinator-dependent crops like macadamia.

## 1. Introduction

Globally, many pollinator-dependent crop systems suffer from pollinator limitation, yet their cultivation is expanding [1,2]. There is increasing recognition that pollinators other than honey bees (*Apis* species), such as native bees, can be important to crop production, either replacing or supplementing services provided by honey bees to protect against pollination shortages [3]. Among these, a small number of eusocial species can also be managed in artificial hives and employed for commercial pollination, notably bumble bees and stingless bees [4]. However, there is often limited information on how to best manage alternative pollinator colonies, particularly in terms of appropriate stocking rates, timing of deployment, and colony spatial distribution. Stingless bees (Apidae: Meliponini) are an important example of an increasingly popular managed pollinator for which such information is often lacking. Developing crop-specific pollinator management strategies can help growers to maximize economic returns, but this requires understanding how various management strategies impact pollinator performance [5].

Using managed colonies of social bees to provide pollination services does not necessarily guarantee that many individuals from those colonies will forage on the target crop. Social bees adjust their foraging behavior in response to the distribution, quantity, and quality of resources within their environment, as well as in response to colony condition and needs, and abiotic factors like ambient temperature [6,7,8]. We know that foragers typically collect resources from a diverse range of plant species to meet colony nutritional requirements [9,10]. However, there is evidence to suggest that social bees can exhibit greater selectivity in their foraging choices when highly profitable resources like mass-flowering trees are available [6,8].

Fidelity to the target crop is a desirable trait in managed pollinators to achieve efficient conspecific pollen transfer. This is especially important for crops that rely heavily on cross-pollination between cultivars to ensure high and consistent yields and quality. The “crop fidelity” of a colony can be defined as the proportion of pollen foragers returning to the hive with pollen from the target crop [11]. Meanwhile, non-target floral resources can include weeds, floral strips, native vegetation, and simultaneously flowering crops. Outside of the target crop flowering period, these other plants provide essential forage for bees, but during crop flowering, they may act as competition [12,13,14]. One method through which crop fidelity can be improved is to introduce colonies at specific times in relation to the flowering period of the target crop. Several studies have suggested that European honey bee (*Apis mellifera* L.) colonies located in a crop prior to its flowering may favor competing floral resources within the local area to which they have already established constancy, particularly if they find them more attractive [15,16,17]. In contrast, introducing colonies once crop flowering has commenced, and in some cases, sequentially adding more colonies, results in more bees foraging on the target crop [15,16,18,19,20,21].

Stingless bee foraging behavior is similar to that of European honey bees in that both taxa are polylectic generalists, display floral constancy (the behavioral tendency of foragers to visit the same plant species in a single foraging bout), and rapidly recruit nestmates to profitable resources [22,23,24]. However, in contrast, stingless bee colonies are smaller (varying in size from hundreds to upwards of 10,000 workers compared to more than 60,000 in a strong honey bee colony), lack the waggle dance communication system, and on average have much shorter foraging ranges [25,26,27]. In orchard environments, the foraging range of *Tetragonula carbonaria* (Smith) ranges from <100 m to around 700 m [28,29], whereas European honey bees can forage up to 14 km from the hive [30]. Additionally, stingless bees exhibit more restricted diurnal foraging activity than honey bees due to higher environmental thresholds for initiating flight. For example, *T. carbonaria* typically begins foraging only once ambient temperatures exceed 18 °C, whereas European honey bees can commence foraging at temperatures as low as 10 °C [31,32]. These factors are likely to result in a different spatial and temporal distribution of stingless bee foragers within landscapes compared to honey bees. However, to date, no studies have examined whether stingless bees exhibit a similar behavior when deployed at different times in relation to crop flowering. In fact, whilst researchers have established that stingless bees are viable pollinators in some Australian cropping systems, few studies have investigated how to optimize their deployment [29].

*Tetragonula carbonaria* is a commercially managed Australian stingless bee species that is being increasingly employed for pollination of macadamia nuts (*Macadamia integrifolia* Maiden & Betche, *M. tetraphylla* L.A.S. Johnson, and hybrids) in New South Wales and Queensland [33]. Macadamia flowers are protandrous and partially self-incompatible and are therefore highly dependent on insect-mediated cross-pollination [34,35]. Two deployment strategies are common in macadamia orchards. In the first, commercially managed colonies are rented and typically introduced at the beginning of crop flowering, remaining on site only for the flowering period. In the second, growers maintain colonies that are located permanently in orchards throughout the year. Whilst these two strategies have various merits and disadvantages, it is particularly important to consider how they impact the delivery of pollination services. For example, permanently located colonies may contribute less to crop pollination than colonies introduced when the crop is in flower if they continue to visit alternative resources in the local landscape. This may have a significant economic impact, as there is evidence that macadamia yields are limited by pollinator deficits [35,36,37]. Consequently, it may be possible to improve pollination and crop yields by using managed colonies of *T*. *carbonaria* more effectively in macadamia orchards.

The main objectives of this study were to (1) establish how the timing of *T. carbonaria* colony deployment during the mass-flowering period of macadamia impacts colony crop fidelity, and (2) determine whether colonies would take advantage of a mass-flowering crop event by increasing the collection of pollen, as opposed to other resources (nectar or resin).

## 2. Results

### 2.1. Macadamia Flowering Levels and Colony Introductions

**Figure 1:**
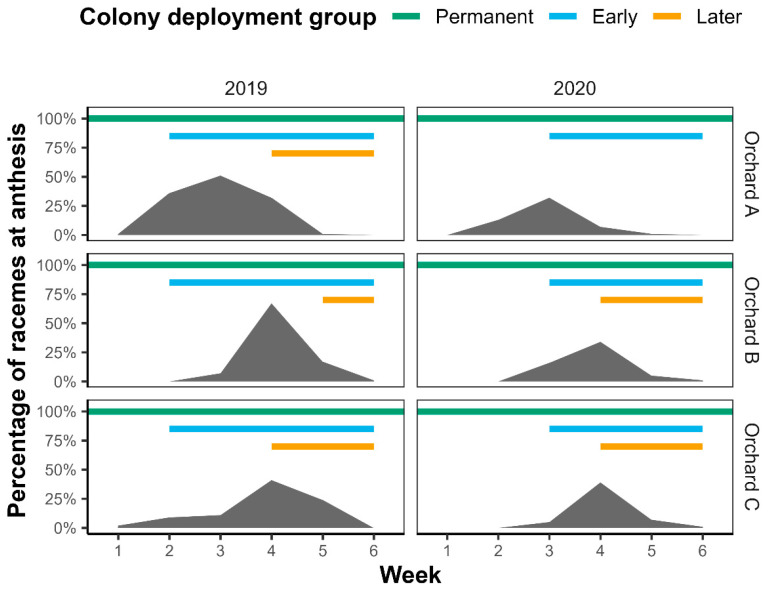
Macadamia flowering levels, measured as the percentage of tagged macadamia racemes at anthesis in each week of the experimental period in Orchards A, B, and C in 2019 and 2020. Colored lines indicate when colony deployment groups (permanent, early, and later) were introduced and how long they were present in the orchards.

Orchards B and C peaked in the number of racemes at anthesis in week four during both years of the experiment, whereas Orchard A flowered earlier and more rapidly, peaking in week three in both years (Figure 1). In 2020, the flowering event occurred slightly later and more rapidly in all orchards, so whilst in 2019, the early colonies were introduced in week two of the experimental period, in 2020, they were introduced in week three. The later colonies were introduced in week four of 2019, except in Orchard B, where colony introduction was delayed until week five. Later colonies were also introduced in week four of 2020, except in Orchard A where they were not introduced as the flowering event ended.

### 2.2. Crop Fidelity

#### 2.2.1. Factors Influencing Crop Fidelity

**Figure A1:**
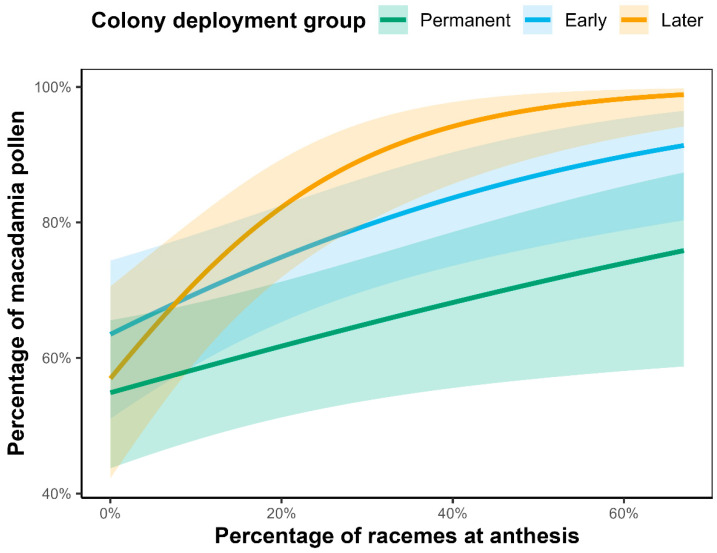
Predicted crop fidelity values (percentage of macadamia pollen) for the three colony deployment groups (permanent, early, and later) across a range of macadamia flowering levels (percentage of tagged racemes at anthesis). Predictions were generated from a GLMM with a beta-binomial distribution. Shaded regions represent 95% confidence intervals for the predicted values.

Colony deployment group had a significant effect on crop fidelity when assessed across the full experimental periods of both years (χ^2^ = 11.363, df = 2, *p* = 0.003), with the two introduced colony groups exhibiting higher crop fidelity compared to the permanent colonies (Table 1). Macadamia flowering levels also had a strong positive relationship with crop fidelity overall (χ^2^ = 22.189, df = 1, *p* < 0.001), but a significant interaction between flowering levels and colony deployment group demonstrated that the scale of response differed between colony groups (χ^2^ = 9.933, df = 2, *p* = 0.007) (Figure A1). The permanent colonies exhibited a more moderate response to flowering levels compared to the introduced colonies. The early colonies had a stronger response but had relatively high crop fidelity even when flowering levels were low. The later colonies had the strongest relationship between crop fidelity and flowering levels, likely due to the synchronicity of their deployment at higher levels of flowering and initial high crop fidelity that subsequently declined as flowering levels dropped. Finally, the proportion of foragers returning to the colony with pollen had a significant negative relationship with crop fidelity (χ^2^ = 5.812, df = 1, *p* = 0.016).

When focusing only on the first week that the introduced colonies were deployed, across all orchards, we found no relationship between crop fidelity and measured macadamia flowering levels (χ^2^ = 0.134, df = 1, *p* = 0.715) and no differences in crop fidelity between the two introduced colony deployment groups (χ^2^ = 0.047, df = 1, *p* = 0.828).

#### 2.2.2. Weekly Differences in Crop Fidelity Between Colony Deployment Groups

**Figure 2:**
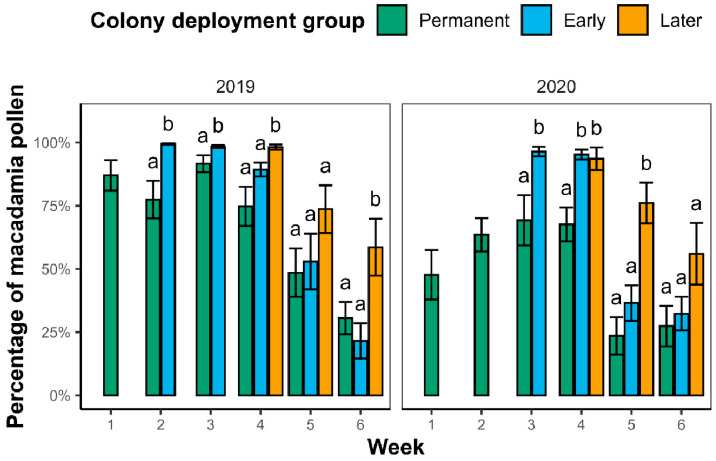
Crop fidelity, displayed as the mean (±SE) percentage of macadamia pollen collected each week of the experimental period, of the three colony deployment groups (permanent, early, and later) in 2019 and 2020. Letters above the bars denote statistically significant differences (*p <* 0.05) between colony deployment groups in that week, determined by Tukey adjusted post hoc pairwise comparisons from generalized linear mixed effect models (GLMMs) with binomial distributions. Later colonies from Orchard B in 2019 are included in the previous weeks to account for their delayed introduction.

In both years, the early colonies brought in a significantly greater proportion of macadamia pollen than the permanent colonies in the first two weeks after their introduction (Figure 2, see Table A1 for full test statistics). In 2019, the later colonies also brought in a significantly greater proportion of macadamia pollen than the permanent and early colonies in the week they were introduced (week four) and the final week of flowering, but not in the penultimate week (five). In 2020, the later colonies brought in a significantly greater proportion of macadamia pollen than the permanent colonies, but not the early colonies, in the week they were deployed. In week five of 2020, the later colonies brought in a significantly higher proportion of macadamia pollen than both the permanent and early colonies, and in week six, there were no significant differences. In summary, the introduced colonies tended to bring in a greater proportion of macadamia pollen than the colonies already present in orchards in the first two to three weeks after deployment.

### 2.3. Pollen Diversity

**Figure 3:**
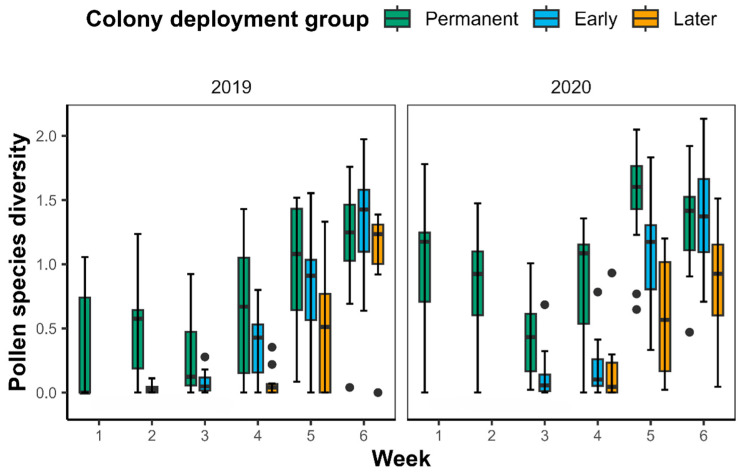
The diversity of pollen species (quantified using the Shannon diversity index) collected each week of the experimental period by the three colony deployment groups (permanent, early, and later) in 2019 and 2020. Later colonies from Orchard B in 2019 are included in the previous weeks to account for their delayed introduction.

**Figure A2:**
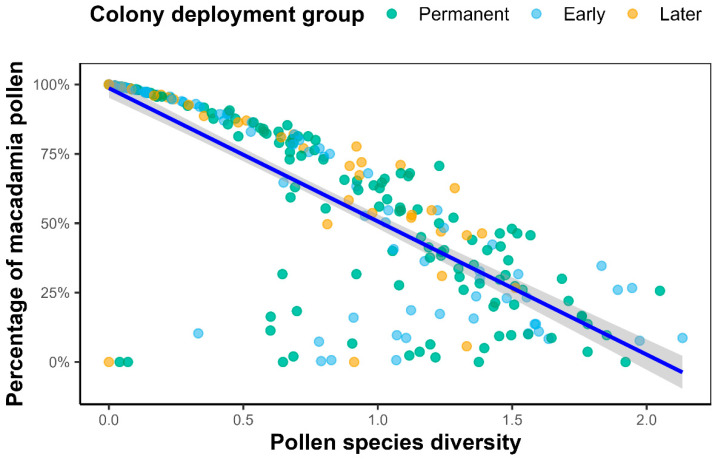
The relationship between pollen species diversity (Shannon diversity index) and crop fidelity (percentage of macadamia pollen). Points are colored by colony deployment group (permanent, early, and later). The fitted line represents a linear model with 95% confidence intervals (shaded region).

Overall, the permanent colonies collected a higher diversity of pollen species than the introduced colonies in the early weeks of the experiment, though we did not see this clearly in all orchards in 2019 (Figure 3). As crop flowering progressed, the introduced colonies increased the diversity of pollen species they collected, such that, in both years, all colony types collected similar (high) diversity of pollen species in the final week of crop flowering. Consistent with this pattern, pollen diversity was strongly negatively correlated with crop fidelity (r = −0.882, *p* < 0.001) (Figure A2). Therefore, low pollen diversity values represent times when incoming pollen was dominated by macadamia pollen.

## 3. Discussion

The overall aim of this study was to investigate how the timing of stingless bee colony deployment, in relation to levels of flowering in macadamia orchards, impacts forager crop fidelity and resource use. We found that the colonies deployed early and later in the flowering period initially displayed higher fidelity to the target crop than the permanent colonies located in orchards prior to flowering. In most cases, the permanent colony foragers were collecting pollen from a diverse range of flowering plants in the local area at the beginning of crop flowering and continued to collect a higher diversity of pollen species throughout the flowering period, though they did take advantage of the mass-flowering event by intensifying macadamia pollen collection. Pollen diversity increased over time in the introduced colonies and was highest in all colonies in the final weeks of the experimental period, when macadamia flowering levels declined. The proportion of foragers collecting pollen was negatively related to crop fidelity, indicating that colonies invested more in pollen foraging when collecting diverse sources of pollen. Our results show that introduced stingless bee colonies display higher crop fidelity than those already present before flowering, particularly prioritizing the mass-flowering macadamia crop when initially deployed, but that bees reduce their crop fidelity over time, probably because colonies explore their surroundings further with time and detect other floral resources.

Eusocial bees naturally collect pollen from a diverse array of plant species within their environment to ensure they collect a balanced ratio of nutrients (i.e., essential amino acids, lipids, and sterols) and to dilute potentially toxic plant compounds [10,38]. This behavior is associated with improved health and performance outcomes when compared to bees fed more restricted or monofloral diets [9,39,40]. Stingless bees, *T. carbonaria* in particular, have been found to maximize intake diversity [41,42,43]. However, this study demonstrates that it is possible to temporarily induce *T. carbonaria* colonies to focus mostly on a single mass-flowering resource by introducing colonies to orchards after the target crop has begun to flower. These initial high crop fidelity values occurred irrespective of the level of flowering within the orchard. In particular, the early introduced colonies brought in large amounts of macadamia pollen even at low flowering levels in early weeks (Figure 2 and Figure A1). This contrasts with some European honey bee studies, where colonies introduced at higher levels of crop flowering collected more crop pollen than those introduced at lower levels [15,16].

Moving colonies to a different environment forces foragers to reorient themselves within their new landscape and locate new floral resources. The idea behind deploying colonies when there are already crop flowers available, and situating them within orchard blocks, is that foragers predominantly find the target crop before other floral resources and establish floral constancy to them. In mass-flowering crops, which provide spatially and temporally concentrated resources, this is possible even at lower flowering levels. It is also possible that our method of assessing macadamia flowering levels was not sensitive enough for a mass-flowering tree crop, and what we classified as low flowering levels may have been perceived by *T. carbonaria* colonies as an abundant resource. Macadamias produce up to two million flowers per tree [44], so even when only a small proportion of racemes were recorded in anthesis, the absolute number of open flowers per tree could constitute a plentiful resource. However, previous studies have shown that the level of flowering intensity within orchards does not strongly correlate with stingless bee visitation rates, suggesting that other factors may play a more significant role in determining *T. carbonaria* visitation, such as proximity to the hive location [29,45,46].

It is worth noting that macadamia appears to be an attractive resource for *T. carbonaria.* Kaluza et al. [43] found that bees collected pollen with very high protein concentrations during the macadamia flowering, indicating that macadamia pollen has a high protein content, a trait that eusocial bees have been shown to purposefully select for [47,48]. Furthermore, macadamia pollen has been detected in hive stores of *T. carbonaria* throughout the year in orchard environments, suggesting it may be stored and accessed beyond the flowering period, as well as used to provision larval cells [41,49]. This likely explains why the permanent colonies in our study, despite displaying a moderate response to flowering levels and maintaining visitation to other floral resources, still displayed relatively high levels of fidelity to the macadamia crop (Figure 2). In 2020, the permanent colonies in all three orchards increased the proportion of macadamia pollen they collected as the mass-flowering event progressed, resulting in a decrease in the diversity of pollen species collected (Figure 3). Similar patterns of reduced floral diversity and increased macadamia pollen in hives kept in orchards have previously been linked to intensified collection of macadamia during its flowering period [41]. In 2019, there were some instances where permanent colonies began to collect more macadamia pollen earlier in the flowering season. In Orchard A, this can likely be attributed to the grower moving the colonies a short distance within the orchard prior to flowering. General variation in the pollen collection behavior of colonies in different orchards and years is also likely explained by site-level differences, including the composition, spatial distribution, and temporal availability of local floral resources, as well as microclimatic conditions in the area of the orchard where colonies were deployed, and internal colony conditions such as brood production and resource demand.

Several studies have found that during periods of greater floral resource availability, stingless bee foragers focus on plants that offer more profitable resources [6,8,50,51]. Stingless bees share the location of profitable floral resources with nestmates to recruit them to that food source [25,52]. Thus, we might expect to see an increase in the proportion of foragers collecting a particular resource, such as pollen, in association with a mass-flowering event. *Tetragonula carbonaria* foragers have been found to predominantly collect pollen in macadamia orchards [53]. However, we found, across all colonies, that a lower proportion of foragers collected pollen when the colonies were bringing in a high proportion of macadamia pollen. This suggests that higher allocations of total forager numbers were actively employed by the colonies to locate and collect diverse pollen sources in order to maximize diversity intake, rather than in response to the mass-flowering event [43].

Recommendations for the optimal timing of European honey bee colony deployment vary across crops [54]. However, the consistent message is the importance of introducing colonies when there is sufficient flower availability to immediately attract foragers to the target crop. Conversely, delaying deployment too long, particularly if no colonies are already present in orchards, means that early opening flowers will not benefit from visits by foragers from rented colonies. This is especially relevant for crops like macadamia, where flowering can occur over a prolonged period [55]. Our findings, and those of others, suggest that a mixed deployment strategy featuring multiple sequential introductions of colonies may be most effective [19,20]. Permanently located and early introduced colonies can ensure that early-opening flowers receive pollination, while later introductions could boost the number of foragers collecting macadamia pollen at peak and late flowering. Stern et al. [20] found that two honey bee colony introduction events, one at 10% flowering and another at peak flowering, increased bee visitation, mobility between rows, and fruit set in pear orchards when compared to only one colony introduction event at 10% flowering. Certainly, in our study, we found that crop fidelity in the introduced colonies was no longer significantly higher than the permanent colonies after two to three weeks, when the colonies began diversifying the types of pollen they collected. It would be worth investigating whether crop fidelity in these colonies could be sustained longer by relocating them to another orchard after two weeks and forcing the foragers to reorient themselves within the landscape again, which would help determine whether naivety to the crop is important when introducing colonies.

Differences in the flowering patterns between the three orchards partly reflect different macadamia cultivars and planting arrangements. The onset, duration, and peak of flowering vary between different cultivars, and this explains why we never recorded 100% when measuring flowering levels [45,55]. Despite our best efforts, predicting the peak of flowering was challenging, so different colony groups were not always introduced at the optimal time, particularly the later colonies (Figure 1). This was especially evident in Orchard A, where trees flowered earlier. However, the fact that the introduced colony groups showed very high crop fidelity irrespective of the level of flowering within the orchard when first deployed suggests that this probably did not affect the results. Growers may face similar issues if attempting to deploy colonies in direct correlation with different stages of flowering, so we suggest that if using a sequential introduction method, additional colonies should simply be introduced two to three weeks after the early colonies, to coincide with the decline in the proportion of foragers collecting macadamia pollen after this length of time. Selecting synchronously flowering cultivars when planting orchards will result in shorter flowering periods, which would be beneficial in reducing the number of colony introduction events required, in addition to improving the chances of cross-pollination occurring.

Crop fidelity can be considered an indirect measurement of colony-level pollination efficiency in macadamia because *T. carbonaria* has been shown to act most efficiently as a pollinator whilst collecting macadamia pollen rather than nectar, during which it makes the most contact with the small stigmatic surface [53,56]. A higher proportion of foragers collecting macadamia pollen suggests greater pollen transfer between macadamia stigmas and thus pollination success. However, it is worth noting that some foragers opportunistically rob pollen from partially opened macadamia flowers, removing pollen without any legitimate contact with the stigma [53]. Heard [53] found that the abundance of *T. carbonaria* and the proportion of individuals engaged in nectar foraging and robbing were positively correlated, which suggests that foragers may switch foraging modes due to increased numbers of conspecific foragers on racemes. However, though we did not quantify it, casual field observations indicated relatively low insect visitation on macadamia racemes during both study years of our study (personal observations of C.E.A.). This suggests colonies experienced little intra- or interspecific competition in this study.

In conclusion, our results indicate that *T. carbonaria* colonies located permanently in macadamia orchards respond moderately to the mass-flowering event by increasing the proportion of macadamia pollen they collect. However, we suggest that sequentially introducing colonies into orchards could be a good method to ensure higher proportions of foragers are collecting macadamia pollen consistently throughout the flowering period, as well as to boost the number of foragers at peak and late flowering. Removing competing resources prior to crop flowering may be another method through which crop fidelity could be improved, particularly with permanently deployed colonies. However, it is important to note that co-flowering plant species can increase the abundance and diversity of wild pollinators [57,58] and are important for *T. carbonaria* colony health outside of crop flowering periods. As cross-pollination is important for macadamia yields and nut quality, future research should also focus on whether individual foragers collecting macadamia pollen visit multiple cultivars in one foraging bout. To test the validity of this methodology more widely, it would be worth repeating this experiment, particularly in crops considered to be less attractive to stingless bees. It would also be beneficial to quantify the impact of deployment timing on pollination outcomes. The timing of colony deployment is just one important aspect of colony management strategies in orchard crop pollination; additional information on the distribution and density of colonies is also required.

## 4. Materials and Methods

### 4.1. Experimental Set-Up

This study was carried out in 2019 and 2020 in the Northern Rivers region of New South Wales, Australia, near the township of Alstonville. Macadamia production is a dominant land use in the region. Observations began on 29 August 2019 and on 28 August 2020, and the experimental period was six weeks long, incorporating the entire macadamia flowering period. The area typically has a subtropical climate with mild, sunny weather throughout this period. We selected three commercial macadamia orchards (labeled Orchards A, B, and C), approximately 20–30 ha in size, which each already had at least four permanently established colonies of the locally occurring stingless bee *T. carbonaria*. The three orchards were separated by a minimum of 1.8 km and a maximum of 8.7 km and were therefore likely subject to broadly similar weather conditions throughout the experimental period. Orchard A was located in Dalwood (−28.888500, 153.410000) and was composed of macadamia cultivars A4, 246, A38, A16, and 660. Orchard B was located in Rous (−28.861200, 153.409500) and was composed of the macadamia cultivars A4, 246, 849, and 344. Orchard C was located in Lindendale (−28.827400, 153.366200) and was composed of macadamia cultivars A4, 246, 344, 849, 816, 359, and A29. All orchards had natural vegetation adjacent to, and interspersed between, blocks of macadamia trees. In addition, they used low pesticide spray regimes combined with IPM strategies.

In each orchard, four of the permanently established *T. carbonaria* colonies were chosen (on the basis that they were near each other and were actively foraging) and designated as the “permanent” colony deployment group. Eight additional colonies were rented from a commercial pollination service provider and collectively labeled as the “introduced” colonies. Four of these colonies were introduced early in the flowering period; the time when commercially managed colonies are typically deployed (designated as the “early” colonies). To understand the importance of the level of flowering when colonies are introduced, four more were then deployed later in the flowering period (“later” colonies). We aimed to introduce these colonies at the peak of flowering, when flowering levels were higher. The introduced colonies were deployed in the same area of the orchard as the permanently deployed colonies (except in Orchard A where they were distributed more widely in 2019) so that foragers from all colonies had access to essentially the same floral resources. All colonies were placed at the edge of macadamia blocks within orchards and could access macadamia trees within the immediate vicinity of the hive (see Figure S1 for colony orientation within each orchard in 2019 and 2020). Orchard C had a large number of additional colonies present within the same orchard (approximately 65, evenly distributed through the orchard), whereas the other two orchards had a smaller number of additional colonies in other areas of the orchard. In 2020, all the sites had an additional eight colonies (deployed at the same time as the early introduction colonies) located within the immediate vicinity of the hives for a separate experiment. There may also have been wild colonies of *T. carbonaria* or *T. hockingsi* in remnant subtropical rainforest patches, windbreaks, and woody corridors in and around the orchards.

**Figure A3:**
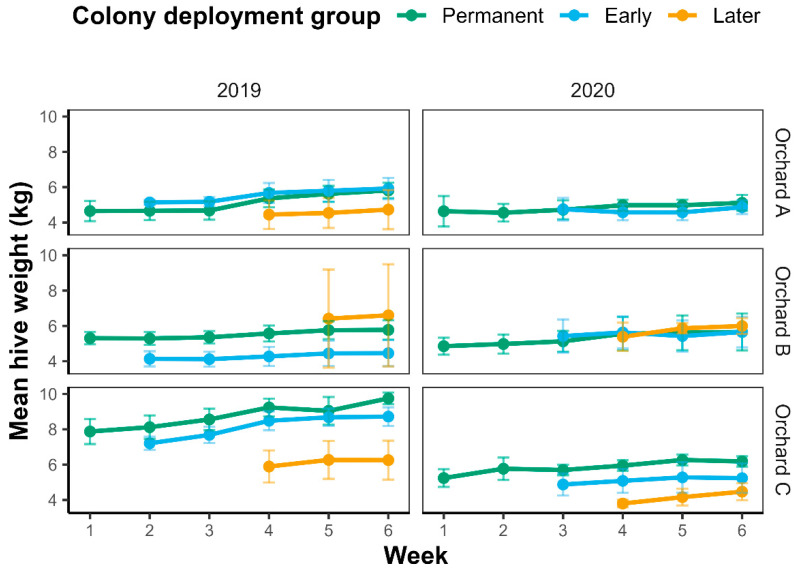
Mean (±SD) hive weight (kg) of colony deployment groups (permanent, early, and later) over the experimental period in orchards A, B, and C in 2019 and 2020.

In the weeks prior to being deployed in orchards, the introduced colonies had not been exposed to macadamia, although they may have been used for macadamia pollination in the previous year. All colonies were housed in standard OATH hives [59] but varied in the time since they had last been split and, consequently, their weight. We weighed the colonies weekly using a digital fishing scale (Pryml Digital Scale, BCF, Strathpine, QLD, Australia) (Figure A3 summarizes the colony weights over the experimental period for each colony deployment group in 2019 and 2020).

### 4.2. Monitoring Macadamia Flowering Levels

To monitor the progression of flowering in each orchard, ten trees within 500 m of the central point of the experimental colonies were randomly selected in each orchard, and three groups of racemes per tree were tagged. This distance was chosen to encompass trees within the foraging range of colonies, and, because the macadamia orchards consisted of a mix of cultivars with differing flowering phenology, selecting trees from within this area helped to capture cultivar diversity (see Figure S1 for tagged tree locations within each orchard in 2019 and 2020). Each group of racemes was made up of 3–10 racemes located at the end of a branch, 1.5 m–2 m from the ground, and distributed evenly around each tree’s circumference. We visited these tagged raceme groups weekly to count the number of individual racemes in each group in three flowering stages: pre-anthesis (elongated racemes with closed green or creamy buds), at anthesis (≥50% of flowers on the raceme had open creamy-white flowers with a straightened style and exposed anthers), and post-anthesis (the perianths of the flowers had withered or fallen away leaving only the style). The proportion of individual racemes that were at anthesis out of all tagged racemes per orchard per week was then used as our weekly macadamia flowering level measure for that orchard.

We began the experimental period just prior to the onset of flowering, when the tagged racemes were at the pre-anthesis stage. This timing allowed us to monitor foraging behavior in permanent colonies before macadamia floral resources became available. We then made decisions about when to introduce colony deployment groups based on the percentage of racemes counted that were at anthesis each week compared to the previous week, as well as following recommendations from growers and our stingless bee pollination service provider. The experiment concluded after six weeks in both years, once all observed racemes had progressed to the post-anthesis stage.

### 4.3. Pollen Sampling

We collected pollen foragers from each colony on one day per week (every 7 ± 3 days) from the beginning of the experimental period for the permanently located colonies, or from when they were deployed in the field in the case of the introduced colonies. To ensure there would be pollen-foraging activity, we chose days that were bright and sunny, with temperatures higher than 18 °C, low wind, and no rain forecasted. Our goal was 20 pollen foragers per colony, with 10 foragers collected at two timepoints per day: in the morning (before 11:00) and in the afternoon (after 13:00). To collect foragers, we temporarily blocked hive entrances and captured returning bees with pollen on their corbiculae individually in 2 mL plastic centrifuge tubes. We did this for as long as was necessary to capture the required number of bees. On the occasions that there were very few pollen foragers, we repeated the process multiple times but abandoned attempts if it was not possible to collect the target number that day. Pollen foragers were then kept chilled whilst in the field, before being transferred to a freezer and stored at −20 °C.

### 4.4. Pollen Preparation and Counts

We pooled the pollen loads from all foragers captured from each colony on the same sampling day, and this sample represented the colony for that week of the flowering period. Out of 308 weekly colony samples, 218 met our target number of 20 foragers, but it was not possible to capture 20 in all cases. We excluded 15 weekly colony samples where no pollen foragers were captured from the analysis. Of the remaining samples, 42 were between 10 and 20, and 30 were lower than 10 (see original data in FigShare with DOI 10.6084/m9.figshare.29062673). In cases where <20 foragers were captured due to low pollen-foraging activity, we still pooled them and assumed this to be a representative sample for the colony in that week. Each sample was homogenized in 100% ethanol (50 µL per bee pollen load, i.e., each pooled colony pollen sample was homogenized in 1 mL of ethanol if derived from 20 foragers).

We took a 100 μL aliquot from each individual colony’s pollen sample per week for microscopic pollen analysis. The pollen samples were acetolysed following the methods detailed by Jones [60] to remove lipids on the pollen surface and reveal identifying structures. Acetolysed pollen samples were then placed on microscope slides together with a drop of fuchsin gelatin and fixed with a cover slip. We examined 300 grains per slide at 400× magnification by moving between 12 intersection points along four vertical and four horizontal transects. Pollen grains were categorized as either macadamia or non-macadamia pollen. Non-macadamia pollen was grouped into morphotypes and, where possible, identified to the lowest taxonomic level possible using pollen atlases. Crop fidelity for each colony per week of each year was calculated as the proportion of pollen grains per microscope slide identified as macadamia compared to other pollen species.

### 4.5. Recording Foraging Activity

Each hive entrance was filmed one day a week (every 7 ± 3 days) on a day pollen forager collection was not performed but under the same weather conditions. The colonies were filmed for 7 min between 11:00 and 12:00 and again from 13:00 to 14:00 with a video camera (HDR-CX405 Handycam, Sony, Tokyo, Japan) on a tripod at the hive entrance. The first minute of all videos was disregarded in case the camera set-up had disturbed the entrance activity. We then watched the first 20 s of each subsequent minute so that we had two minutes of foraging activity data in total. As we watched, we recorded the number of foragers entering the hive with pollen, resin, or with no visible resources (assumed to be either a returning nectar or water forager, or bees returning from depositing waste or orientation flights). Foraging data from the two video recording time points per day were averaged for that day for statistical analysis.

### 4.6. Statistical Analyses

Statistical analyses were performed with the software R (version 4.4.3) [61]. All data were visualized using the ggplot2 package [62]. Generalized linear mixed models (GLMMs) were fitted using the glmmTMB package [63], the significance of explanatory variables was assessed with Type II Wald tests using the Anova function in the car package [64], and post hoc pairwise comparisons were made using the emmeans package with Tukey’s post hoc adjustment for multiple comparisons [65]. When crop fidelity (the proportion of macadamia pollen compared to other pollen species) was the response variable, it was entered as a two-column binomial vector using the cbind function, where the number of macadamia pollen grains counted was entered as successes and the number of non-crop pollen grains as failures. All interaction terms were initially included in the models but were removed if they were not significant. Model fit was assessed using the DHARMa package to test for overdispersion, deviations from normality, and homoscedasticity [66].

#### 4.6.1. Factors Influencing Crop Fidelity

To understand the relationship between crop fidelity, pollen-foraging effort, and macadamia flowering levels across the colony deployment groups, we fitted a GLMM using a beta-binomial distribution and logit link function. Crop fidelity was the response variable, and the fixed effects were the macadamia flowering level (the proportion of racemes at anthesis each week per orchard), colony deployment group, and proportion of foragers returning to the colony carrying pollen. Colony ID nested within the orchard and year were included as random effects. To account for the significant interaction between colony deployment group and macadamia flowering level, we made pairwise comparisons between deployment groups at a representative value of flowering (14.3%) using Tukey-adjusted post hoc tests. We visualized the relationship by generating predicted values using estimated marginal means from the fitted GLMM using the emmeans package [65], with predictions made over the range of observed flowering levels.

To determine whether macadamia flowering levels had a significant effect on crop fidelity in the introduced colonies when they were initially deployed, or whether deployment in the mass-flowering crop itself was more influential, we filtered the data to obtain a subset that only included introduced colonies in their first week of deployment and constructed a GLMM with a beta-binomial distribution and logit link function. Crop fidelity was the response variable, and the fixed effects were macadamia flowering levels and colony deployment group, while colony ID nested within the orchard was included as a random effect.

#### 4.6.2. Weekly Comparisons of Crop Fidelity Between Colony Deployment Groups

To investigate how crop fidelity differed between the colony deployment groups in each individual week of the experimental period per year (excluding weeks where only permanent colonies were present), we divided the data into subsets for each week per year. The later colonies were introduced to Orchard B a week later than Orchards A and C in 2019, so we included them in the model for the previous weeks. We constructed GLMMs with binomial distributions and logit link functions for each individual week. Crop fidelity was the response variable, colony deployment group was the fixed effect, and colony ID nested within the orchard was included as a random effect to account for differences between individual colonies at different locations. Post hoc pairwise comparisons were made using Tukey-adjusted post hoc tests.

#### 4.6.3. Pollen Diversity

We calculated the diversity of pollen collected by each colony per week using the Shannon diversity index via the diversity function from the vegan package [67] based on counts of individual pollen morphotypes per sample. To examine the relationship between crop fidelity and pollen diversity, we performed a Spearman correlation test.

## Figures and Tables

**Table 1:** Results of Tukey-adjusted post hoc pairwise comparisons comparing crop fidelity between colony deployment groups. To account for the significant interaction between colony deployment group and macadamia flowering levels, we made pairwise comparisons between deployment groups at a representative value of flowering (14.3%). Significant differences (*p* < 0.05) are in bold.

Pairwise Comparison	Estimate	SE	Df *	z-Ratio	*p*
Permanent–Early	−0.541	0.189	Inf	−2.863	**0.012**
Permanent–Late	−0.779	0.237	Inf	−3.291	**0.003**
Early–Late	−0.238	0.252	Inf	−0.947	0.611

* Estimates are based on asymptotic z-tests using the standard normal distribution; therefore, degrees of freedom are effectively infinite.

## Data Availability

The original data presented in the study and R code used for data cleaning, analysis, and visualization are openly available in FigShare with the DOI 10.6084/m9.figshare.29062673.

## Supplementary Materials

The following supporting information can be downloaded at: https://www.mdpi.com/article/10.3390/plants14152313/s1, Figure S1: The spatial arrangement of tagged trees, colonies in each deployment group, and vegetation types for each orchard in 2019 and 2020.

## Appendix A

**Table A1:** Comparison of crop fidelity between colony deployment groups (permanent, early, and later) for each week of the experimental period in years 2019 and 2020 based on post hoc pairwise comparisons using z-tests based on asymptotic inference (Tukey-adjusted) from generalized linear mixed effect models (GLMMs) with binomial distributions. Later colonies from Orchard B in 2019 are included in the previous weeks to account for their delayed introduction. Significant differences (*p* < 0.05) are in bold.

Year	Week	Pairwise Comparison	Estimate	SE	Df *	z-Ratio	*p*
2019	2	Permanent–Early	−4.77	0.692	Inf	−6.902	**<0.001**
	3	Permanent–Early	−1.58	0.69	Inf	−2.286	**0.022**
	4	Permanent–Early	−0.901	0.760	Inf	−1.186	0.416
		Permanent–Later	−3.458	0.825	Inf	−4.191	**<0.001**
		Early–Later	−2.557	0.848	Inf	−3.016	**0.007**
	5	Permanent–Early	−0.326	1.35	Inf	−0.242	0.968
		Permanent–Later	−2.259	1.37	Inf	−1.644	0.227
		Early–Later	−1.933	1.38	Inf	−1.401	0.340
	6	Permanent–Early	0.393	0.773	Inf	0.509	0.867
		Permanent–Later	−2.085	0.888	Inf	−2.349	**0.049**
		Early–Later	−2.478	0.899	Inf	−2.755	**0.016**
2020	3	Permanent–Early	−3.84	1.08	Inf	−3.557	**<0.001**
	4	Permanent–Early	−2.69	0.788	Inf	−3.415	**0.002**
		Permanent–Later	−3.59	0.988	Inf	−3.638	**<0.001**
		Early–Later	−0.90	1.010	Inf	−0.893	0.645
	5	Permanent–Early	−0.921	0.614	Inf	−1.051	0.29
		Permanent–Later	−3.671	0.700	Inf	−5.244	**<0.001**
		Early–Later	−2.750	0.718	Inf	−3.829	**<0.001**
	6	Permanent–Early	−0.897	0.842	Inf	−1.065	0.536
		Permanent–Later	−1.845	0.978	Inf	−1.886	0.143
		Early–Later	−0.948	0.969	Inf	−0.979	0.59

* Estimates are based on asymptotic z-tests using the standard normal distribution; therefore, degrees of freedom are effectively infinite.
