# Supplementary material for: The Impact of Colony Deployment Timing on Tetragonula carbonaria Crop Fidelity and Resource Use in Macadamia Orchards
Source: Plants (Basel). 2025 Jul 26;14(15):2313. doi: 10.3390/plants14152313 (PMC12348977; doi:10.3390/plants14152313)
Supplement: Supplementary file 1 [file plants-14-02313-s001.zip › Allison_et_al_MDPI_Plants_2025_Figure S1.pdf]

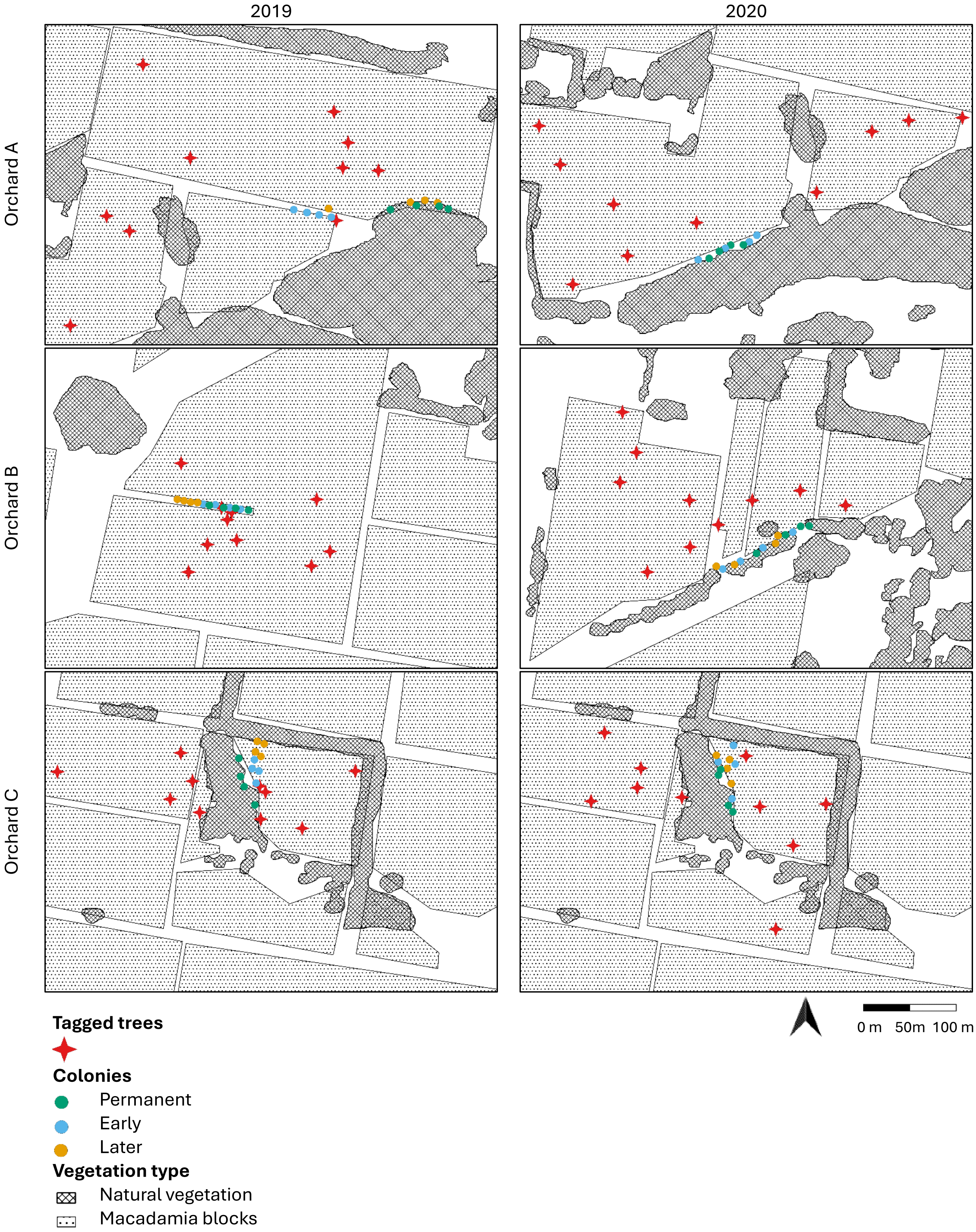

Figure S1. The spatial arrangement of tagged trees, colonies in each deployment group, and vegetation types for each orchard in 2019 and 2020.
